# Atherosclerosis and Endometriosis: The Role of Diet and Oxidative Stress in a Gender-Specific Disorder

**DOI:** 10.3390/biomedicines11020450

**Published:** 2023-02-03

**Authors:** Michela Cirillo, Flavia Rita Argento, Monica Attanasio, Matteo Becatti, Irene Ladisa, Claudia Fiorillo, Maria Elisabetta Coccia, Cinzia Fatini

**Affiliations:** 1 Department of Experimental and Clinical Medicine, University of Florence, 50134 Florence, Italy; 2 Centre for Assisted Reproductive Technology, Division of Obstetrics and Gynaecology, Careggi University Hospital, 50134 Florence, Italy; 3 Department of Clinical and Experimental Biomedical Sciences, University of Florence, 50134 Florence, Italy

**Keywords:** cardiovascular disease, atherosclerosis, endometriosis, Mediterranean Diet, lifestyle, risk factors, oxidative stress, gender medicine

## Abstract

*Background*: Accelerated atherosclerosis in patients with endometriosis has been hypothesised, and lifestyle improvement might control cardiovascular risk. We explored cardiometabolic markers and oxidative stress and evaluated the effects of the Mediterranean Diet (MD) in modulating these markers. *Methods*: In this prospective study, we included 35 women with endometriosis. At baseline (T0) and after 3 (T1) and 6 (T2) months from the start of the diet, we investigated cardiometabolic parameters, lifestyle and oxidative stress. *Results*: After a 3-month intervention with MD, we observed a significant reduction in total cholesterol (*p* = 0.01) and LDL-c (*p* = 0.003). We observed at T1 an increase in B12 and E vitamins, folate and zinc. After 6 months, zinc (*p* = 0.04) and folate (*p* = 0.08) increased in comparison to T0. A reduction in homocysteine from T0 to T1 (*p* = 0.01) was found. After 3 months, an increase in Rapid Assessment of Physical Activity tool 1 (RAPA) (*p* < 0.001) and RAPA 2 was observed (*p* = 0.009). We observed high levels of oxidative stress markers at baseline. After 6 months of MD, a significant improvement in lymphocyte Reactive Oxygen Species (ROS) (*p* < 0.001) and total antioxidant capacity was observed (*p* = 0.02). *Conclusions*: The improvement of lifestyle, and in particular the Mediterranean dietary intervention, allowed the improvement of the metabolic and oxidative profile and overall health-related quality of life.

## 1. Introduction

Atherosclerosis represents a multifocal and progressive systemic disease of the arterial wall. The pathogenesis of atherosclerosis is linked to the interaction of known and emerging risk factors [1]. The key element in the pathogenesis of this systemic disorder is endothelial dysfunction (ED), which is characterised by an altered endothelium-dependent vasodilation and by a specific state of “endothelial activation”. ED promotes all stages of atherogenesis as a proinflammatory, proliferative and procoagulant. Therefore, ED may reflect a vascular phenotype with atherogenic potential. In ED pathogenesis, reactive oxygen species (ROS) hyperproduction and oxidative stress appear to have a relevant role, being closely associated with both traditional and emerging atherosclerotic risk factors [2]. ED is a reversible disorder, and therapeutic strategies aimed at controlling cardiovascular risk factors may translate a “sick” endothelium into a “health” endothelium. In the context of cardiovascular diseases, we cannot ignore a gender-specific approach, so it is necessary to analyse not only the prevalence of traditional risk factors and their different impacts in relation to gender, but also those emerging risk factors that are more common or exclusive in the female gender, such as endometriosis.

Endometriosis is a chronic, debilitating disease affecting roughly 10% (190 million) of reproductive-age women globally [3]. It is associated with pelvic pain and infertility and is characterised by endometrial-like tissue outside the uterus [4].

Endometriosis and atherosclerosis are traditionally viewed as distinct entities, with endometriosis affecting young reproductive-age women, while atherosclerosis is an ageing-related process. The link between endometriosis and cardiovascular diseases is increasingly being studied and has been partly explained by systemic inflammation, with potentially underlying genetic similarities that, in turn, increase the risk of atherosclerosis, coronary artery disease and cardiovascular morbidity and mortality [5]. Chronic inflammation, oxidative stress, endothelial dysfunction, and cellular proliferation are common hallmarks of both atherosclerosis and endometriosis [6,7,8]. Altered expression of antioxidants and abnormal lipid profiles have also been identified as risk factors for cardiovascular diseases and have similarly been found to be altered in women with endometriosis.

Oxidative stress, increased inflammatory cytokines and mast cell activation are considered relevant steps in the pathophysiology and progression of the disease [9]. Retrograde menstruation is likely to carry into the peritoneal cavity several well-known inducers of oxidative stress, such as erythrocytes, apoptotic endometrial tissue, and cell debris, in addition to pelvic macrophages [10]. Chronic inflammation and oxidative stress are not restricted to the peritoneal cavity and induce a state of systemic subclinical inflammation. Findings from the literature evidenced the beneficial effects of food’s natural phytocomponents and dietary patterns, rich in flavonoids and polyphenols, in oxidative diseases [11]. In particular, experimental data reported that hydroxytyrosol, a natural compound of olive oil, exerted anti-inflammatory and antioxidant effects and decreased endometriotic cyst diameter, area and volume [9].

Endometriosis has also been associated with an atherogenic lipid profile, notable for increased LDL, non-high-density lipoprotein and triglycerides, and decreased HDL levels [12,13]. The most convincing evidence was provided by Mu et al. in Nurses’ Health Study II, with a 25% increased risk of developing hypercholesterolaemia in endometriosis and a 22% increased risk of developing laparoscopically confirmed endometriosis in women with hypercholesterolaemia [14]. It would signal the need for targeted prevention, and early detection guidelines for chronic and life-threatening diseases in women with endometriosis are needed. In this context, the role of nutrition in determining the establishment and progression of endometriosis has recently become a topic of interest and several observational studies have investigated certain nutrition habits and lifestyles as risk factors for endometriosis [15,16].

The role of diet in endometriosis has gained more attention since it has been observed that diet can affect several processes that are involved in endometriosis, including inflammation, prostaglandin metabolism, and oestrogen activity [17]. To date, it is evident that this topic is characterised by an extreme paucity of scientific data and by an extreme variability in the results obtained. Moreover, it is important to note the difference between the role of diet in the risk of developing endometriosis and a dietary intervention with the aim of suppressing endometriosis-related symptoms.

Lifestyle improvement by adopting Mediterranean dietary patterns, an evidence-based nutritional model for the prevention of cardiovascular disease, might also represent a future non-pharmacological therapeutic intervention for controlling cardiovascular risk in women with endometriosis. No data are available concerning the role of the Mediterranean Diet in influencing subclinical atherosclerosis, and in turn cardiovascular risk, in endometriosis. Based on the above-mentioned observations, the aim of this study is to explore cardio-metabolic, endothelial, oxidative stress and inflammatory markers correlated with the atherosclerotic process, and to evaluate the effects of Mediterranean Diet intervention in modulating these markers.

## 2. Materials and Methods

### 2.1. Study Design

In this prospective study, we investigated 90 Caucasian women with endometriosis referred to the Internal Medicine Clinic at the Centre for Assisted Reproductive Technology, Division of Obstetrics and Gynaecology of Careggi University Hospital, Florence, from March 2020 to May 2022. All women were referred to the Endometriosis Centre of Careggi University Hospital, Florence, a third-level centre for endometriosis treatment. In all patients, the diagnosis of endometriosis was confirmed by diagnostic imaging (US or MRI) and/or laparoscopy performed by gynaecologists expert in this field. Pre-existing atherothrombotic disorders, hypertension, diabetes, autoimmune diseases, renal failure, obesity, pregnancy and chronic illnesses that are known to affect gastrointestinal absorption of nutrients (celiac disease, Crohn’s disease, ulcerative colitis, or cystic fibrosis) represented exclusion criteria. Women participating or who had participated in a weight loss treatment and nutrition programme in the last 3 months were excluded. Therefore, we included 35 women with endometriosis of reproductive age in the study. At the first evaluation, all women were in therapy with oestrogen-progestins or progestins. The baseline assessment included demographic information and cardiovascular risk factors. Moreover, at the first visit, we focused on the current nutritional habits of endometriosis patients before the start of the diet. We administered a validated questionnaire to evaluate accurate data about dietary intake and adherence to the Mediterranean Diet [18,19].

During the subsequent 10 days after the first visit, the participants completed a food diary giving a detailed description of each food consumed, the time of consumption, and amount, using household measures, in order to capture their habitual diet and stool habit assessment. After the evaluation of the food diary, we developed a personalised tailored nutrition Mediterranean Diet plan agreed upon with the patient to achieve the best compliance. The energy requirements were calculated for each woman. The diet consisted of 50–55% carbohydrate, 25–30% total fat (≤10% saturated fat) and 15–20% protein. Each woman was provided with a 1-week menu plan as well as information on the food groups that could be included and those that should be avoided.

During the study, 3 clinical evaluations were performed: at the baseline, before the start of dietary treatment (T0), 3 months after the start of the dietary intervention (T1), and after a further 3 months (6 months from the beginning) (T2). At baseline (T0) and after 3 (T1) and 6 (T2) months from the start of the diet, we evaluated:

- Coagulative profile, including VIII Factor (FVIII), von Willebrand Factor (vWF), vitamin profile (Folate, B12, B6, E) and zinc, endothelial markers (homocysteine, Lp(a)), lipid and glucose profile, liver panel enzymes, hs-CRP;
- Blood redox status (oxidative stress markers such as, lipid peroxidation markers, plasma total antioxidant capacity and blood leukocyte subpopulation ROS production).

At each clinical evaluation, anthropometric parameters (weight, height, BMI, waist and hip circumference, waist to hip ratio) were measured. Compliance with the Mediterranean Diet was evaluated during follow-up visits using a validated adherence score [19].

The investigation conformed to the principles outlined in the Declaration of Helsinki. The Local Ethics Committee (Azienda Ospedaliero-Universitaria Careggi) approved the original study (Reference: 21140).

### 2.2. Assessment of Cardiovascular Risk Factors

We assess women’s vascular profiles by collecting information about the family history of CVDs, defined as the history of CVDs in first-degree relatives <55 years of age in men and <65 years in women [20]. During the clinical evaluation, traditional (dyslipidaemia, overweight and abdominal fat, smoking habit, sedentary behaviour), prevalent and sex-related (migraine with aura, history of negative obstetric events, such as recurrent-2-pregnancy loss, recurrent implantation failure and placenta-mediated pregnancy complications) CV risk factors were also investigated. Weight was measured in underwear and without shoes with a mechanical column scale with a stadiometer (SECA). According to the WHO criteria, underweight was defined when the BMI was <18.5 Kg/m^2^, normal weight when the BMI was between 18.5 and 24.99 Kg/m^2^, and overweight when the BMI was 25 Kg/m^2^. In order to evaluate abdominal fat, anthropometric parameters were also measured. Hip circumference was measured at the widest point over the buttocks, and waist circumference was measured midway between the inferior margin of the lowest rib and the iliac crest in the horizontal plane at the end of normal expiration. A waist circumference of 80 cm was considered a marker of increased CV risk according to Alberti et al. [21]; waist to hip ratio (WHR) was obtained by dividing the waist circumference by hip circumference, and values 0.85 were considered a marker of increased cardiovascular risk [22]. To limit measurement bias, all anthropometric parameters were performed by the same operator. Dyslipidaemia was defined according to the European Society of Cardiology (ESC) guidelines [23]. Hyperhomocysteinaemia was defined as a pathological condition of excessive plasma homocysteine level (>13 μmol/L). Smokers were defined as current or recent (ex-smokers who stopped less than 5 years earlier) smokers. The diagnosis of migraine with aura was performed by a physician according to The International Classification of Headache Disorders 3rd edition [24]. The Rapid Assessment of Physical Activity (RAPA) questionnaire was administered to assess the level of physical activity. RAPA evaluates a wide range of physical activity levels, from sedentary to vigorous activity, as well as strength and flexibility training [25].

### 2.3. Laboratory Analysis

Lipid profile (lipoprotein (a), total cholesterol, HDL-c, LDL-c, triglycerides), homocysteine and vitamins (folate, vitamin B12, vitamin B6, vitamin E), zinc, hs-CRP, glucose and coagulative profiles, including FVIII and vWF, were determined at the University Careggi Hospital Laboratory.

### 2.4. Assessment of Blood Redox Status

#### 2.4.1. Sample Collection

Blood samples were collected in Vacutainer tubes containing 0.109 mol/L buffered trisodium citrate (1:10) or EDTA (0.17 mol/L). After centrifugation (1500× *g* for 15 min at 4 °C), plasma was collected and used for experiments or stored at −80 °C for further analysis. Oxidative Stress markers were measured in the Laboratory of Oxidative Medicine of the Department of Clinical and Experimental Biomedical Sciences “Mario Serio” University of Florence, Italy.

#### 2.4.2. ROS Assessment in Leukocytes (Lymphocytes, Monocytes and Granulocytes) by Flow Cytometry Analysis

The dichlorofluorescein diacetate H2DCFDA probe (Invitrogen, Carlsbad, CA, USA) was used for intracellular ROS evaluation, as previously reported [26,27].

#### 2.4.3. Plasma Lipid Peroxidation Estimation (ALDetect Lipid Peroxidation Assay)

Plasma lipid peroxide levels were estimated using an ALDetect Lipid Peroxidation assay (BML-AK170-Enzo Life) as previously reported [28]. The results are expressed as equivalent of MDA (nmol/mL).

#### 2.4.4. Plasma Total Antioxidant Capacity Estimation

The ORAC (oxygen radical absorbance capacity) assay was performed as previously reported [29]. Plasma total antioxidant capacity was calculated using the standard curve based on Trolox concentration. The results are expressed as Trolox equivalents (μM) and then normalised for the protein concentration.

### 2.5. Statistical Analysis

The sample size was calculated starting from historical data [30] and estimating an improvement (reduction) in High-sensitive C Reactive Protein (hs-CRP) of 0.70 with an SD of 1.4. Assuming a power of 0.80 and α setting of 0.05, the number was estimated for 33 subjects.

The results were expressed as median (range) or mean, as appropriate. Categorical variables were presented in terms of frequencies and percentages. Statistical analysis was performed using McNemar to analyse dichotomous variables and the Wilcoxon rank-sum test for continuous variables for paired data. Unpaired Mann–Whitney U-tests were performed to compare the independent groups. Repeated measures ANOVAs were used to examine the mean differences in the subgroups of women who completed three evaluations. The Spearman (rho) test was used to estimate the correlation between the variables. Differences were considered statistically significant if *p* < 0.05. Statistical analysis was performed using IBM SPSS Statistics 28 for Windows (SPSS, Chicago, IL, USA).

## 3. Results

A total of 35 participants completed the first phase of the intervention. Sixteen women completed the entire study, and 19 women did not complete phase 2 (T2): two of them became pregnant in the meantime, while for what concerns, the remaining 17, less than three months have passed from T1.

About 37% of the study population had a high level of education (>13 years of education).

From gynaecologists’ medical reports, we obtained information about the phenotype of endometriosis; in particular, 17.1% of women had Deep Infiltrating Endometriosis (DIE) and 17.1% had a mixed phenotype (DIE + Ovarian Endometrioma).

The baseline demographic characteristics of the population studied are shown in Table 1. The patients in the study collective were 31 (21–46) years of age. The evaluation of traditional CV risk factors at baseline evidenced that about 11% of women had a BMI between 25–29.99 kg/m^2^ while about 23% of women were underweight. In about 26% of women, we evidenced values of waist circumference above 80 cm.

Dyslipidaemia was present in more than 50%, in particular, in 40% of women with high levels of LDL-c.

In framing women’s risk profiles, we also considered family history of CVD, and we observed that more than 25% had a family history of CVD. As concerns migraine, which is known as a stronger and more prevalent risk factor in women, we observed a high prevalence of migraine with aura (11.4%).

Finally, by analysing the composition of the diet through a food diary at T0, we observed a daily intake of macronutrients, with carbohydrates accounting for 48% of dietary energy. Carbohydrate intake was lower than desirable for the Mediterranean dietary pattern (50–55%), while sugar consumption was high, if we consider that the baseline evaluation showed that only 14.3% of the patients assumed 2–3 portions of fruit a day. Indeed, we assumed that the quantity of total sugar intake was mainly due to the consumption of added sugars. The consumption of fibre was lower when compared to the 15 g/1000 Kcal/day recommended by the LARN [31], and this can also be attributed to the low percentage of women who regularly consumed fruit and vegetables. Moreover, we observed that the consumption of whole grains was not too frequent either.

Fat consumption exceeded 30% of energy intake and, specifically, the median intake of saturated fatty acids was 10.5%, with values ranging from 6.6% up to 16.6%.

### 3.1. Biochemical Parameters

Biochemical parameters at T0, including coagulative profile, vitamins, homocysteine, liver and renal function, and fasting glucose, are shown in Table 2.

**Figure 1:**
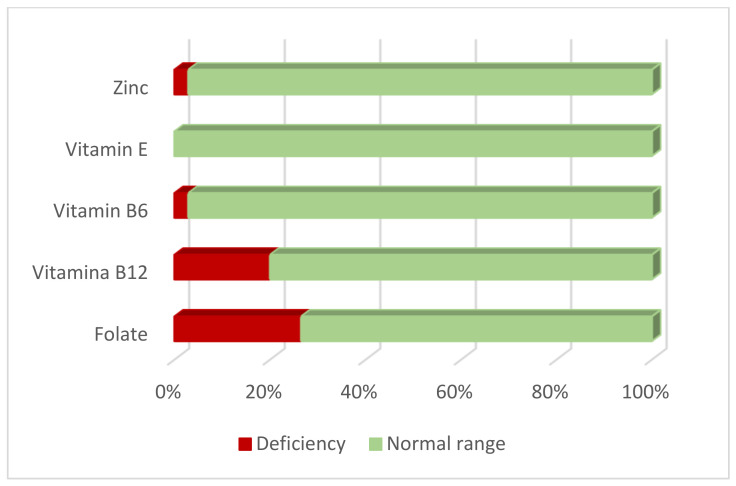
Status of Zinc and Vitamins (E, B6, B12 and Folate) in women with endometriosis.

The overall frequency of folate and vitamin B12 deficiencies was estimated to be 26.5% and 20%, respectively (Figure 1). The frequency of elevated homocysteine levels was 8.6% (>13 μmol/L).

We observed no vitamin E deficiency and only 2.9% of women had reduced levels of vitamin B6 and zinc.

### 3.2. Changes in Anthropometric and Biochemical Parameters

We evaluated anthropometric parameters in women from T0 to T2 and we evidenced no significant differences in the BMI, waist circumference and WHR between T0 vs. T1 and T0 vs. T2.

We analysed changes in anthropometric parameters according to BMI at baseline; therefore, we considered changes in women that at T0 were underweight, women with normal BMI and women overweight. Table 3, Table 4 and Table 5 show the changes in the anthropometric parameters at T1 and T2. In particular, in underweight women (Table 4), we observed at T1 a significant increase in BMI (*p* = 0.04), while in overweight women (Table 5), we evidenced at T1 a reduction in waist circumference and WHR, even if not significant (*p* = 0.06). Of relevance, 3 women underweight at T0 had normal BMI at T1 and T2.

At T0, all the anthropometric parameters were inversely related to the Mediterranean Diet adherence score, albeit without statistical significance, whereas this correlation was significant at T1 (BMI rho = −0.40, *p* = 0.02; Waist rho = −0.52, *p* = 0.001; WHR rho = −0.44, *p* = 0.009), indicating an inverse association more evident when adherence to the Mediterranean Diet increased.

We further evaluated changes in anthropometric parameters by performing repeated measures analysis in a subgroup of women (*n* = 16) who completed three evaluations (Table 6).

Regarding the lipid profile, we observed a significant reduction in the level of total cholesterol (*p* = 0.01), and in particular in LDL-c levels (*p* = 0.003). This trend of reduction in LDL-c seems to also be present at T2, even if not significant, probably due to the small sample size (Table 7). Therefore, at T1, only 14.3% of women (vs. 31.4% at T0) had total cholesterol levels above 200 mg/dL, and 20% of women (vs. 40% at T0) had LDL-c levels above 116 mg/dL. There were no significant changes in the prevalence of hypertriglyceridaemia or low HDL-c levels.

**Figure 2:**
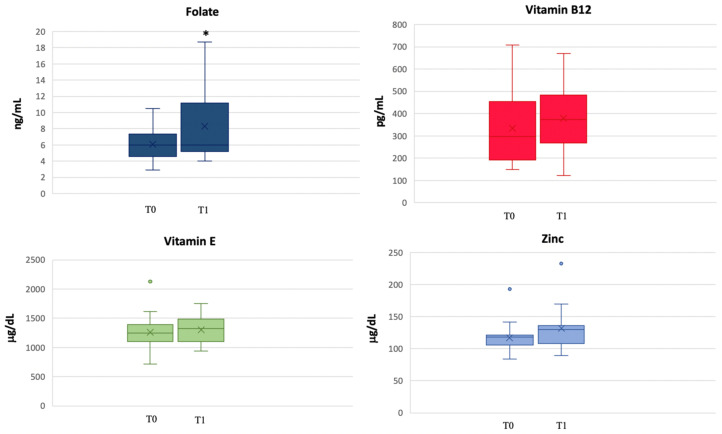
Changes in folate, vitamin B12, vitamin E and zinc concentrations from T0 to T1. * *p* < 0.05.

Moreover, we analysed the vitamin profile, and we observed an increase in the levels of B12 and E vitamins, folate and zinc (Figure 2). In particular, there was a statistically significant increase in folate levels from T0 to T1 (*p* = 0.01). After six months, in the 16 patients who completed the clinical evaluation at T2, zinc (T0 vs. T2 *p* = 0.04) and folate (T0 vs. T2 *p* = 0.08) levels increased in comparison to T0. Finally, at T1, we observed a reduction in the percentage of patients with B12 vitamin deficit (20% at T0 vs. 5.9% at T1), whereas all women with folate deficit had already corrected this condition.

**Figure 3:**
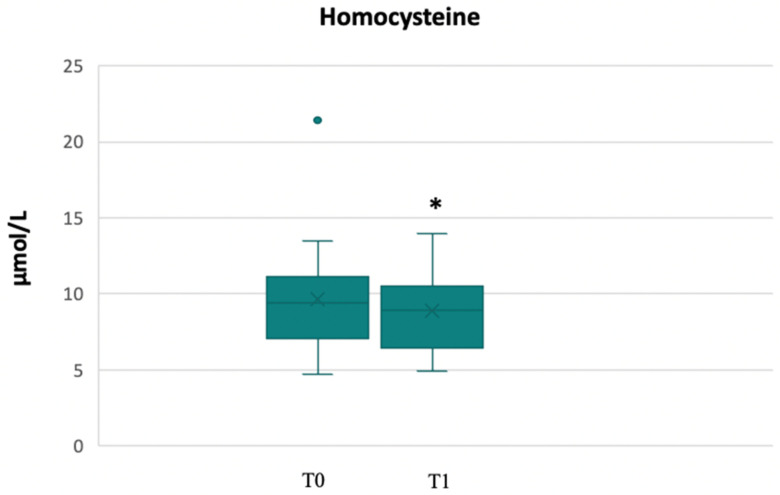
Changes in homocysteine levels from T0 to T1. * *p* < 0.05.

A reduction in homocysteine levels from T0 to T1 (9 μmol/L vs. 7.9 μmol/L, *p* = 0.01), probably due to an improvement in vitamins regulating its metabolism (folate, B12 vitamin, and B6 vitamin), was found (Figure 3). Indeed, two out of three patients with homocysteine levels above 13 μmol/L, had normalised their levels at T1. In just one patient, the level of homocysteine was slightly beyond the range of normality.

Regarding inflammatory status, hs-CRP levels were beyond the normal range in 14.3% of the patients. At T1, we found a slight reduction, even if not statistically significant, in this parameter (1 mg/L vs. 0.8 mg/L). At T2, in the sample that completed the study, we observed a median value of hs-CRP of 0.6 (0.01–3.4).

### 3.3. Lifestyle Changes

**Figure 4:**
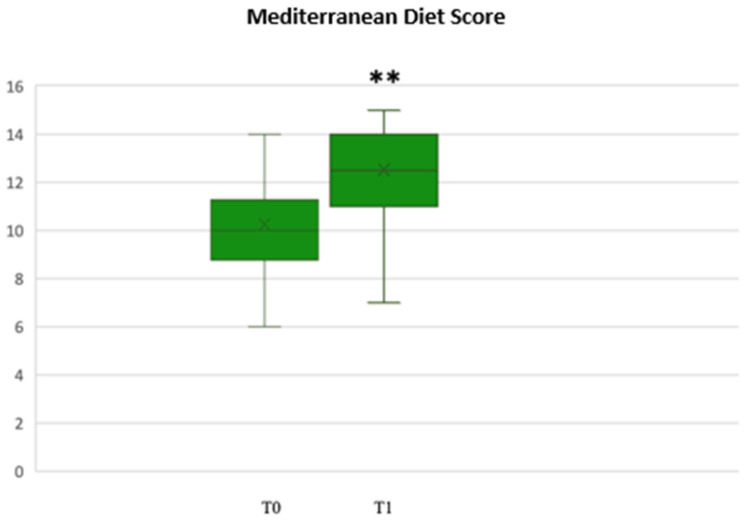
Mediterranean score adherence at T0 and T1. ** *p* < 0.001.

Through the analysis of the Mediterranean Diet adherence score at the end of the first phase, we found a statistically significant increase in the median adherence score (T0 vs. T1, *p* < 0.001) (Figure 4), which was still significantly higher at T2, with a median value of 13 (8–15) (T0 vs. T2, *p* < 0.001).

The nutrition evaluation took into account the daily water intake that resulted very low in some patients, with a median value of 1.5 L (0.25–3). At T1, we found an important increase to 1.75 L (0.25–4), although not statistically significant (*p* = 0.07). This improvement may be due to the season in which each woman did their T1 visit (spring or summer rather than fall or winter).

**Figure 5:**
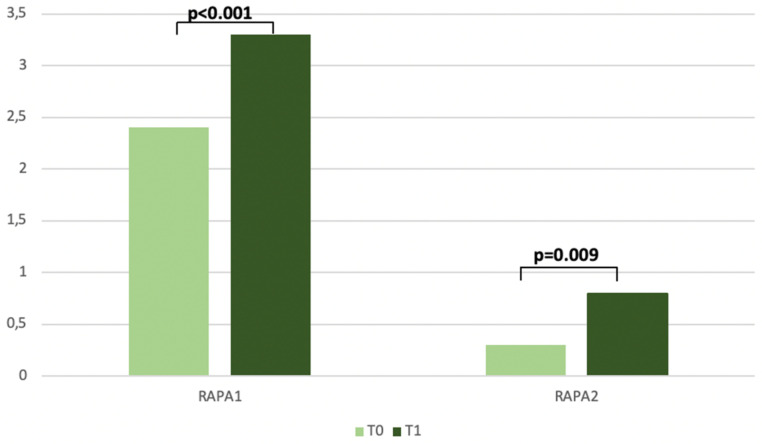
Changes in Rapid Assessment of Physical Activity from T0 to T1.

The physical activity levels were evaluated with the Rapid Assessment of Physical Activity (RAPA) tool. At baseline, the median levels were very low for both RAPA 1 (Aerobic) 2.4 (1–6) and RAPA 2 (Strength and Flexibility) 0.3 (0–2). After the first 3 months from the beginning of the intervention, the increase was statistically significant for both RAPA 1 (*p* < 0.001) and RAPA 2 (*p* = 0.009). Regarding the patients who concluded the study, RAPA 1 and RAPA 2 significantly increased from T0 to T2 (*p* = 0.01 and *p* = 0.04, respectively) (Table 6). In general, a high prevalence of patients has enhanced their aerobic activities (fast walking, aerobics classrooms and swimming) as well as stretching or yoga for flexibility (Figure 5).

### 3.4. Oxidative Stress

We evaluated blood global redox status by assessing leukocyte intracellular ROS production, plasma lipid peroxidation, and plasma total antioxidant capacity. We observed significant alterations compared to the normal range observed in a population of healthy age-matched women (as previously published) [32], in particular as regards plasma lipid peroxidation (at T0 was increased in 85.7% of women), level of total antioxidant capacity (at T0 was reduced 51.4% of women), Neutrophil-ROS production (at T0 was increased in 68.6%), Monocyte-ROS production (at T0 was increased 77.1% of women) and Lymphocyte-ROS production (at T0 was increased in 62.9% of women).

**Figure 6:**
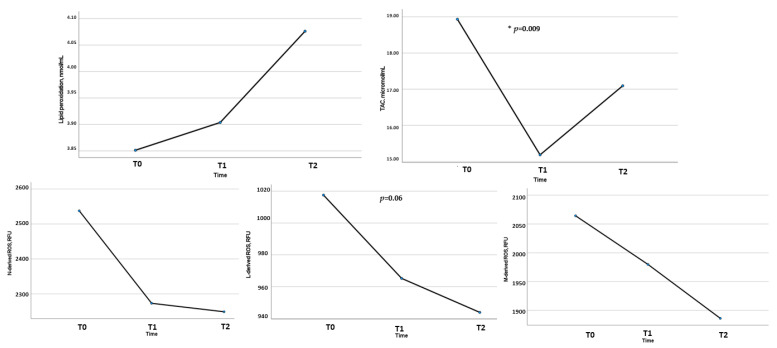
Changes in Oxidative Stress Parameters from T0 to T2 (*n* = 16). N indicates neutrophil; L, lymphocyte; M, monocyte; RFU, relative fluorescence unit; ROS, reactive oxygen species; TAC, total antioxidant capacity. * *p* < 0.05.

We did not observe significant changes between T0 and T1, but there was an improvement between T0 and T2 (Table 8). In particular, by analysing ROS production and plasma antioxidant capacity in patients who concluded the study compared to the initial sample, we observed a statistically significant reduction in Lymphocyte-ROS (*p* < 0.001) and an increase in total antioxidant capacity (*p* = 0.02). We also observed this result by analysing ROS production and plasma antioxidant capacity from T0 to T2 in 16 women who concluded the study (Figure 6). Moreover, considering the relation between adherence to the Mediterranean dietary pattern and oxidative stress profile, we observed at T2 (after 6 months) a significant inverse correlation between leukocyte ROS production and adherence to the Mediterranean Diet (Neutrophil-ROS: rho = −0.53, *p* = 0.04; Monocyte-ROS: rho = −0.66, *p* = 0.007; Lymphocyte-ROS: rho = −0.55, *p* = 0.03).

## 4. Discussion

This study aimed to evaluate the role of the Mediterranean Diet and lifestyle in improving the cardiovascular risk profile of women with endometriosis and investigated common mechanisms shared by atherosclerosis and endometriosis. The aetiopathogenesis of endometriosis is a multifactorial process that determines the development of an extremely heterogeneous disease. Several hypotheses have been suggested; nevertheless, none is able to completely explain its pathogenesis and all its different clinical features. Evidence suggests that immune cells, adhesion molecules, extracellular matrix metalloproteinase and pro-inflammatory cytokines activate the peritoneal microenvironment, leading to differentiation, adhesion, proliferation and survival of ectopic endometrial cells [33]. Systemic chronic inflammation, oxidative stress and proatherogenic lipid profile are the main mechanisms involved in the development and progression of atherosclerosis and represent the possible link with endometriosis. It could be useful to bridge the gap between comorbidities and atherosclerotic burden in young women with endometriosis by integrating knowledge of endometriosis with clinical assessment of internal medicine [13].

Data from the Nurses’ Health Study II [34] suggested that endometriosis is inversely associated with early adult BMI. A recent experimental study addressed the causal relationship involving BMI, metabolic status and endometriosis in a mouse model [35]. Mice with experimentally induced endometriosis were found to display lower body weight than the controls, leading to the conclusion that endometriosis is causal to the loss of body weight and body fat. Metabolic dysfunction and obesity usually co-exist, but disrupted metabolic status can also arise independently of body weight. In our study, we excluded obese women in order to limit confounding factors on inflammation markers. We observed that about 11% of women were overweight and about 26% had waist circumference higher than 80 cm, a well-established independent predictor of morbidity and mortality [36]. On the other hand, we found that about 23% of women were underweight, thus possibly underlining the already known relationship between lower BMI and endometriosis. Of interest, we observed after the 3-month intervention a significant increase in BMI in underweight women and a significant reduction in waist circumference and WHR in overweight women.

Concerning the link between endometriosis and dyslipidaemia, few studies have investigated the lipid profile in women with endometriosis [14]. In our study, we observed that more than 50% of women suffered from dyslipidaemia. Our findings are in keeping with Melo et al. [12]; moreover, we provided information about another marker of atherosclerosis and endothelial dysfunction, strictly related to lipid metabolism, such as Lipoprotein (a), that resulted in alterations in about 26% of our study population. Based on these observations, we evaluated the eating habits at baseline through food diaries, and we observed a percentage of fat intake above 30%, with more than 10% saturated fatty acids, as previously reported in other studies [37]. Regarding the other macronutrient intake at baseline, women with endometriosis showed lower relative carbohydrate and protein intake. Data from the literature showed that specific fatty acids seem to influence the risk for endometriosis. A high intake of long-chain omega-3 fatty acids decreases the risk, whereas a high intake of trans-unsaturated fat seems to favour the development of endometriosis and inflammatory processes, being associated with increased menstrual pain and autoimmune and hormonal disorders [38]. Furthermore, murine studies have shown a significant influence of an elevated fat intake (45% of daily calorie requirements come from fat) on endometriosis lesions, with an increase in pro-inflammatory cytokines and oxidative stress [39]. Because inflammation plays a pivotal role in the pathogenesis and progression of endometriosis, regulation or monitoring of quantitative and qualitative fat intake might be recommended for disease treatment. At the end of the 3-month intervention with Mediterranean Diet, we observed that this dietary pattern was very effective in reducing total and LDL cholesterol levels.

These data reinforce the previous evidence regarding the healthy effects of the Mediterranean Diet since the development of a less atherogenic LDL phenotype could be a possible explanation for some of the cardioprotective benefits of this dietary pattern [40]. Data from the literature evidenced that the peritoneal fluid of women with endometriosis is characterised by high lipoprotein levels, particularly LDL-c, which generates oxidised lipid components in a macrophage-rich inflammatory milieu [41]. To date, LDL oxidation represents a major cause of endothelial injury, responsible for leukocyte and macrophage migration [42] and induction of inflammatory cytokines, thus favouring internalisation of oxidised LDL particles and contributing to the formation of atheromatous plaques. LDL-c oxidation is a crucial event in the development of atherosclerosis, with low LDL levels reducing the risk of major events in patients with CVD [43]. It is well known that LDL level reduction prevents oxidation, as recognised by European and American societies of cardiology guidelines [44]. Furthermore, LDL oxidation is not the sole initiator of inflammation, as the imbalance between oxidants and antioxidants also plays an important role in the atherogenesis process.

In the evaluation of biohumoral parameters, besides the lipid profile, we evaluated blood levels of vitamin E and zinc, beyond B6, B12 vitamins and folates that are known to be linked to homocysteine levels. Our endometriosis patients showed significantly lower levels of vitamin B12 and folate at baseline, just as higher levels of homocysteine were found in about 9% of women. Hyperhomocysteinemia is most commonly caused by B-vitamins’ deficiency, especially folate, B6 and B12. Elevated homocysteine promotes atherosclerosis through increased oxidative stress, impaired endothelial function, and thrombosis induction [45]. Since vitamin B12 is primarily included in animal products, the lower intake of protein (unbalanced diet) in comparison to fat in endometriosis patients could be responsible for this observation. Similarly, folate levels were presumably linked to an irregular vegetables’ intake, as observed in the eating habits at baseline. At the end of the 3-month intervention, we observed the efficacy of the Mediterranean Diet in reducing homocysteine concentration and in increasing folate and vitamin B12 levels. Since B-vitamins are assumed to have positive effects on endometriosis and inflammation processes in general, the relevance and influence of lower vitamin B12 or folate intake needs further investigation [46].

With respect to antioxidant vitamins, the mean vitamin E level in our study population fell within the normal range. The main function of vitamin E is to act as a structural antioxidant, in particular at the membrane level. In general, a deficiency of this vitamin exacerbates the inflammatory response and impairs both cellular and humoral immunity. Vitamin E, a lipid-soluble antioxidant, can also delay or prevent oxidative stress-induced diseases. Indeed, it may be considered a neutralising agent against endometriotic cell-derived ROS [47,48]. Our results evidenced, after 3 months of intervention, higher levels of vitamin E, as well as zinc, an intracellular signalling molecule with anti-inflammatory properties that has an essential role in oxidative stress and immune functions, inhibiting free radical production [49]. Findings from the literature have reported that lower zinc levels were seen in women with endometriosis in comparison to controls [50]. We observed zinc deficiency in just one patient, but at the same time, a progressive and significant increase after the intervention period started was present in the whole sample. Regarding inflammatory status, it is well-known that hs-CRP represents a predictor of all-cause mortality associated with endothelial dysfunction and possibly reflects the development of atherosclerosis [51]. In the present study, about 14% of women at baseline showed increased hs-CRP compared to the normal range, and after 3 months, a slight reduction was observed. In our previous study performed in women with endometriosis, we evidenced higher mean levels of hs-CRP, which is possibly due to the larger sample size and older age of the subjects investigated [13]. Inflammation together with macrophages, lipid peroxides, and pain-inducing prostaglandins play a vital role in the pathophysiology of endometriotic pain, as suggested by previous studies [52,53].

### Oxidative Stress

An increasing body of evidence has suggested that oxidative stress is closely associated with atherosclerotic progression and plaque instability [54]. Oxidative stress is considered a major mechanism involved in endothelial dysfunction and ROS production is associated with cardiovascular risk factors such as hypertension, diabetes, smoking and dyslipidaemia [55]. In the artery wall, ROS generation promotes and activates several pathological pathways involved in atherosclerosis, including lipid oxidation, expression of adhesion molecules, stimulation of vascular smooth muscle cell proliferation and migration, cell apoptosis and activation of metalloproteinase [56,57]. Cellular ROS accumulation induces irreversible damage to cellular components, such as proteins, lipids and DNA, generally leading to necrosis. The connection between endometriosis and ROS production has been widely accepted and deeply studied [10]. In this study, we investigated for the first time a potential link between the Mediterranean Diet and systemic redox status in women with endometriosis. Our findings reveal that women with endometriosis show signs of oxidative stress in the blood at baseline. We observed that Mediterranean Diet intervention contributed to a significant improvement only after 6 months in lymphocyte ROS production (which resulted significantly reduced compared to baseline levels) and total antioxidant capacity (which resulted increased compared to baseline levels), while, after 3 months, possibly due to the short intervention time, no significant modifications were evident. Of interest, we observed an inverse correlation between ROS production and adherence to the Mediterranean Diet. This datum is in keeping with Dai et al. [58], who showed a robust association between adherence to the Mediterranean Diet and lower oxidative stress, supporting the cardioprotective effects of this dietary pattern performed on monozygotic and dizygotic twin pairs.

Other dietary interventions (such as gluten-free diet, low-Ni diet and low FODMAP diet) evaluated the correlation between adherence to diet and improvement of symptoms in endometriosis, but not the effect on oxidative stress [59]. Moreover, Ott et al. demonstrated that the Mediterranean Diet might lead to symptom relief in patients suffering from endometriosis, possibly improving endometriosis-associated pain symptoms via various mechanisms, such as an anti-inflammatory effect [60].

There is growing evidence of the involvement of oxidative stress in female reproduction, particularly in endometriosis. The studies on this topic are highly heterogeneous and aimed to evaluate the effects of dietary supplementation on oxidative stress in endometriosis [59]. Data from studies on mouse models for endometriosis suggest that assuming antioxidant supplements, such as hydroxytyrosol, could be beneficial for endometriosis, as women with endometriosis have immunological processes and elevated pro-inflammatory cytokines, like that of other chronic inflammatory diseases [9]. Therefore, further studies are needed to elucidate the potential role of antioxidant supplementation on ROS in women with endometriosis.

In this study, there are some limits to consider together with the very promising data generated so far. First, the limited duration of the study and the limited number of participants who completed the whole intervention, as enrolment was performed during the COVID-19 pandemic, induced the Italian government to enforce restrictions on outdoor activities and collectively quarantined the population. Second, we are aware that 6 month intervention represents a limited period that permits only to suggest a possible interpretation of the given results. Studies with a larger population and a longer duration are necessary to confirm these intriguing results. Third, in our study, all participants were Caucasian; thus, the findings may not be generalisable to other racial and ethnic groups. Finally, no information concerning intima media thickness (IMT), a structural parameter of subclinical atherosclerosis, was provided. Nevertheless, IMT reflects structural vascular damage that takes a longer time to realise [61]. Notwithstanding these limitations, this study has several strengths. The current research represents a comprehensive analysis of multiple domains of vascular health and various parameters analysed in the same group of participants at different time points. Moreover, another strength is the prospective design of the study, which permits the investigation of events that will take place after the study has been initiated. A second benefit of a prospective study is the ability to account for exposures that vary over time in a given individual.

## 5. Conclusions

Our findings provide evidence of an unfavourable cardiovascular profile, as well as of unhealthy lifestyle habits in women with endometriosis, with results appearing of clinical relevance due to the mean age of the study population. Lifestyle improvement, and in particular the Mediterranean dietary intervention, allowed to ameliorate the metabolic and oxidative profile and provided a substantial improvement in the overall health-related quality of life.

Comprehensive and interdisciplinary approaches to managing endometriosis and interventions aiming to increase the education and disease awareness of patients are mandatory in order to provide prompt and accurate diagnosis and treatment and to allow progress in the discovery of possible effective pharmacological and non-pharmacological interventions. In the field of gender medicine, the evaluation of the cardiovascular risk profile cannot neglect a gender-specific approach, since women’s health is burdened by exclusive risk factors, such as endometriosis, currently the object of attention by experts in several disciplines. To date, the innovative contribution of this study is represented by the use of a non-pharmacological approach, such as the Mediterranean Diet. This dietary pattern is known to be a cornerstone in the prevention of cardiovascular risk but has limited clinical evidence in the gynaecological field, and specifically in the modulation of common pathogenetic mechanisms correlating endometriosis with subclinical atherosclerosis. This study, by promoting research, prevention, and treatment of this high social impact disease, could help improve the quality of life and long-term cardiovascular health of young women suffering from endometriosis.

The results of this study will help to identify useful indicators in order to define the emerging role of endometriosis in the development of the early atherosclerotic process that exposes women of reproductive age to higher cardiovascular risk. Our results can contribute not only to scientific research, providing further evidence to the ongoing discussion about the correlation between atherosclerosis and endometriosis and the role of the latter as a gender-specific cardiovascular risk factor, but also to suggest intervention programmes aimed at promoting a healthy lifestyle in women with endometriosis.

Further studies are needed to confirm our results and to deeply explore specific aspects related to different hormonal therapies, adherence to the Mediterranean Diet and endometriosis phenotype.

## Figures and Tables

**Table 1:** Baseline Characteristics of the Study Population.

Variables	Patients (*n* = 35)
Age, y, median (range)	31 (21–46)
Weight, kg, median (range)	56 (41–83)
BMI, kg/m^2^, median (range)	20.6 (16.4–29.8)
Underweight (<18.5 kg/m^2^), *n* (%)	8 (22.9)
Overweight (25–29.99 kg/m^2^), *n* (%)	4 (11.4)
Waist circumference ≥80 cm, *n* (%)	9 (25.7)
WHR ≥ 0.85, *n* (%)	5 (14.3)
Smoking habit, *n* (%)	9 (25.7)
Migraine, *n* (%)	15 (42.9)
Migraine with aura, *n* (%)	4 (11.4)
Dyslipidaemia, *n* (%)	18 (51.4)
Total cholesterol > 200 mg/dL, *n* (%)	11 (31.4)
LDL cholesterol > 116 mg/dL, *n* (%)	14 (40)
HDL cholesterol < 48 mg/dL, *n* (%)	4 (11.4)
Triglycerides > 150 mg/dL, *n* (%)	1 (2.9)
Lipoprotein (a) > 300 mg/L, *n* (%)	9 (25.7)
History of negative obstetric events, *n* (%)	1 (2.9)
Family history of Endometriosis, *n* (%)	6 (17.1)
Family history of CVD, *n* (%)	9 (25.7)

BMI indicates Body Mass Index; WHR, Waist to hip ratio; LDL, low-density lipoprotein; HDL, high-density lipoprotein; CVD, cardiovascular disease.

**Table 2:** Biochemical Parameters at T0.

Blood Biomarkers	Patients (*n* = 35)
Homocysteine μmol/L	9 (4.7–21.4)
Folate, ng/mL	5.6 (1–14.4)
Vitamin B12, pg/mL	310 (99–709)
Vitamin B6, μg/L	11.6 (4.4–30.2)
Vitamin E, μg/dL	1285 (720–2612)
Zinc, μg/dL	114 (76–193)
Fasting glucose, mg/dL	84 (71–120)
AST, U/L	15 (9–33)
ALT, U/L	11 (7–56)
Creatinine, mg/dL	0.71 (0.57–0.93)
hs-CRP, mg/L	1 (0.2–9)
PT, %	95 (78–115)
aPTT, seconds	30 (26.9–36.1)
Fibrinogen, mg/dL	282.5 (162–560)
Factor VIII, %	115.1 (62.8–265.6)
Factor von Willebrand, %	107 (53.6–205)

Data are reported as median (range). ALT indicates alanine aminotransferase; AST, aspartate aminotransferase; PT, Prothrombin time; aPTT, Activated Partial Thromboplastin Time.

**Table 3:** Anthropometric parameter changes in women with normal BMI.

Variables	T0	T1	T2	*p*	*p*
	(*n* = 23)	(*n* = 23)	(*n* = 10)	(T0 vs. T1)	(T0 vs. T2)
BMI, kg/m^2^	21.3 (18.6–24.3)	21.5 (18–24.1)	21.9 (19.6–23.5)	0.8	0.02
Waist circumference, cm	75 (67–88)	75 (67–84)	72.5 (67–82)	0.4	0.06
WHR	0.78 (0.73–0.88)	0.79 (0.71–0.87)	0.77 (0.70–0.83)	0.3	0.03

Data are reported as median (range). BMI indicates Body Mass Index; WHR, Waist to hip ratio.

**Table 4:** Anthropometric parameter changes in women underweight.

Variables	T0	T1	T2	*p*	*p*
	(*n* = 8)	(*n* = 8)	(*n* = 4)	(T0 vs. T1)	(T0 vs. T2)
BMI, kg/m^2^	18 (16.4–18.4)	18.1 (17–20.4)	18.3 (16.8–20.4)	0.04	0.1
Waist circumference, cm	66 (58–72)	66.5 (60–72)	67 (66–70)	0.2	0.5
WHR	0.79 (0.73–0.84)	0.78 (0.73–0.84)	0.78 (0.76–0.81)	0.5	0.2

Data are reported as median (range). BMI indicates Body Mass Index; WHR, Waist to hip ratio.

**Table 5:** Anthropometric parameter changes in women overweight.

Variables	T0	T1	T2	*p*	*p*
	(*n* = 4)	(*n* = 4)	(*n* = 2)	(T0 vs. T1)	(T0 vs. T2)
BMI, kg/m^2^	27.9 (27–29.8)	27 (26.3–30.1)	28 (26–30.1)	0.5	-
Waist circumference, cm	95 (91–102)	91.5 (86–101)	97.5 (93–102)	0.06	-
WHR	0.90 (0.85–0.94)	0.88 (0.83–0.93)	0.90 (0.86–0.93)	0.06	-

Data are reported as median (range). BMI indicates Body Mass Index; WHR, Waist to hip ratio.

**Table 6:** Anthropometric parameters, vitamins and lifestyle changes from T0 to T2 (*n* = 16).

Variables	T0	T1	T2	*p*
	(*n* = 16)	(*n* = 16)	(*n* = 16)	
BMI, kg/m^2^	21.7	21.6	21.5	0.8
Waist circumference, cm	76.6	75.6	75.4	0.1
WHR	0.80	0.79	0.78	0.008
Folate, ng/mL	5.9	7.8	9.1	0.02
Vitamin B12, pg/mL	344.7	357.7	376.4	0.6
Vitamin E, µg/dL	1506	1486	1481	0.9
Zinc, µg/dL	110.2	106	123.8	0.09
Homocysteine, µmol/L	9.2	8.4	9	0.6
Mediterranean Diet Score	9.8	12.3	12.4	<0.001
RAPA 1	2.5	3.1	3.9	0.01
RAPA 2	0.1	0.9	0.7	0.04

Data are reported as mean. BMI indicates Body Mass Index; WHR, Waist to hip ratio; RAPA Rapid Assessment of Physical Activity.

**Table 7:** Changes in lipid profile.

Variables	T0	T1	T2	*p*	*p*
	(*n* = 35)	(*n* = 35)	(*n* = 16)	(T0 vs. T1)	(T0 vs. T2)
Total cholesterol, mg/dL	183 (142–288)	176 (144–254)	189 (138–248)	0.01	0.2
HDL-c, mg/dL	63 (41–99)	62 (40–89)	69 (42–94)	0.3	0.6
LDL-c, mg/dL	111 (57–191)	101.5 (72–158)	92 (66–159)	0.003	0.1
Triglycerides, mg/dL	70 (31–191)	67 (25–269)	51 (34–221)	0.4	0.4

Data are reported as median (range). LDL-c, low-density lipoprotein cholesterol; HDL-c, high-density lipoprotein cholesterol.

**Table 8:** Changes in Oxidative Stress Parameters.

Variables	T0	T1	T2
	(*n* = 35)	(*n* = 35)	(*n* = 16)
Lipid peroxidation, nmol/mL	3.9 (2.6–8.7)	4 (2.1–6.1)	3.9 (2.7–5.9)
TAC, μmol/mL	16.8 (9.3–27.8)	15.1 (11.3–19.4)	17.9 (9.4–21.5) *
M-derived ROS, RFU	1853 (980–3387)	2068 (1113–3658)	1595 (1273–3646)
N-derived ROS, RFU	2216 (1167–4537)	2457 (1186–3745)	1940 (1376–4387)
L-derived ROS, RFU	884 (510–1612)	985 (574–1940)	822 (650–1765) **

Data are reported as median (range). N indicates neutrophil; L, lymphocyte; M, monocyte; RFU, relative fluorescence unit; ROS, reactive oxygen species; TAC, total antioxidant capacity. * *p* < 0.001; ** *p* = 0.02.

## Data Availability

Not applicable.

## References

[1] Faxon D.P., Fuster V., Libby P., Beckman J., Hiatt W.R., Thompson R.W., Topper J.N., Annex B.H., Rundback J.H., Fabunmi R.P. (2004). Atherosclerotic Vascular Disease Conference: Writing Group III: Pathophysiology. Circulation.

[2] Bonetti P.O., Lerman L.O., Lerman A. (2003). Endothelial dysfunction: A marker of atherosclerotic risk. Arter. Thromb. Vasc. Biol..

[3] Becker C.M., Bokor A., Heikinheimo O., Horne A., Jansen F., Kiesel L., King K., Kvaskoff M., Nap A., Petersen K. (2022). ESHRE guideline: Endometriosis. Hum. Reprod. Open.

[4] Giudice L.C. (2010). Clinical practice. Endometriosis. N. Engl. J. Med..

[5] Roifman I., Beck P.L., Anderson T.J., Eisenberg M.J., Genest J. (2011). Chronic inflammatory diseases and cardiovascular risk: A systematic review. Can. J. Cardiol..

[6] Burney R.O., Giudice L.C. (2012). Pathogenesis and pathophysiology of endometriosis. Fertil. Steril..

[7] Davignon J., Ganz P. (2004). Role of endothelial dysfunction in atherosclerosis. Circulation.

[8] Libby P., Buring J.E., Badimon L., Hansson G.K., Deanfield J., Bittencourt M.S., Tokgözoğlu L., Lewis E.F. (2019). Atherosclerosis. Nat. Rev. Dis. Primers.

[9] Cordaro M., Salinaro A.T., Siracusa R., D’Amico R., Impellizzeri D., Scuto M., Ontario M., Interdonato L., Crea R., Fusco R. (2021). Hidrox^®^ and Endometriosis: Biochemical Evaluation of Oxidative Stress and Pain. Antioxidants.

[10] Carvalho L.F., Samadder A.N., Agarwal A., Fernandes L.F., Abrão M.S. (2012). Oxidative stress biomarkers in patients with endometriosis: Systematic review. Arch. Gynecol. Obstet..

[11] Tarozzi A., Angeloni C., Malaguti M., Morroni F., Hrelia S., Hrelia P. (2013). Sulforaphane as a potential protective phytochemical against neurodegenerative diseases. Oxid. Med. Cell. Longev..

[12] Melo A.S., Rosa-e-Silva J.C., Rosa-e-Silva A.C., Poli-Neto O.B., Ferriani R.A., Vieira C.S. (2010). Unfavorable lipid profile in women with endometriosis. Fertil. Steril..

[13] Cirillo M., Coccia M.E., Petraglia F., Fatini C. (2021). Role of endometriosis in defining cardiovascular risk: A gender medicine approach for women’s health. Hum. Fertil..

[14] Mu F., Rich-Edwards J., Rimm E.B., Spiegelman D., Forman J.P., Missmer S.A. (2017). Association Between Endometriosis and Hypercholesterolemia or Hypertension. Hypertension.

[15] Parazzini F., Viganò P., Candiani M., Fedele L. (2013). Diet and endometriosis risk: A literature review. Reprod. Biomed. Online.

[16] Darling A.M., Chavarro J.E., Malspeis S., Harris H.R., Missmer S.A. (2013). A prospective cohort study of Vitamins B, C, E, and multivitamin intake and endometriosis. J. Endometr. Pelvic Pain Disord..

[17] Vennberg Karlsson J., Patel H., Premberg A. (2020). Experiences of health after dietary changes in endometriosis: A qualitative interview study. BMJ Open.

[18] Sofi F., Macchi C., Abbate R., Gensini G.F., Casini A. (2013). Mediterranean diet and health status: An updated meta-analysis and a proposal for a literature-based adherence score. Public Health Nutr..

[19] Sofi F., Dinu M., Pagliai G., Marcucci R., Casini A. (2017). Validation of a literature-based adherence score to Mediterranean diet: The MEDI-LITE score. Int. J. Food Sci. Nutr..

[20] Piepoli M.F., Hoes A.W., Agewall S., Albus C., Brotons C., Catapano A.L., Cooney M.-T., Corrà U., Cosyns B., Deaton C. (2016). European Guidelines on cardiovascular disease prevention in clinical practice: The Sixth Joint Task Force of the European Society of Cardiology and Other Societies on Cardiovascular Disease Prevention in Clinical Practice (constituted by representatives of 10 societies and by invited experts) Developed with the special contribution of the European Association for Cardiovascular Prevention & Rehabilitation (EACPR). Eur. Heart J..

[21] Alberti K.G.M.M., Zimmet P., Shaw J., IDF Epidemiology Task Force Consensus Group (2005). The metabolic syndrome—A new worldwide definition. Lancet.

[22] World Health Organization (2011). Waist Circumference and Waist to Hip Ratio: Report of a WHO Expert Consultation, Geneva, 8–11 December 2008.

[23] Mach F., Baigent C., Catapano A.L., Koskinas K.C., Casula M., Badimon L., Chapman M.J., De Backer G.G., Delgado V., Ference B.A. (2020). 2019 ESC/EAS Guidelines for the management of dyslipidaemias: Lipid modification to reduce cardiovascular risk. Eur. Heart J..

[24] Headache Classification Committee of the International Headache Society (IHS) (2013). The International Classification of Headache Disorders, 3rd edition (beta version). Cephalalgia.

[25] Topolski T.D., LoGerfo J., Patrick D.L., Williams B., Walwick J., Patrick M.B. (2006). The Rapid Assessment of Physical Activity (RAPA) among older adults. Prev. Chronic Dis..

[26] Becatti M., Marcucci R., Gori A.M., Mannini L., Grifoni E., Liotta A.A., Sodi A., Tartaro R., Taddei N., Rizzo S. (2016). Erythrocyte oxidative stress is associated with cell deformability in patients with retinal vein occlusion. J. Thromb. Haemost..

[27] Whittaker A., Sofi F., Luisi M.L.E., Rafanelli E., Fiorillo C., Becatti M., Abbate R., Casini A., Gensini G.F., Benedettelli S. (2015). An organic khorasan wheat-based replacement diet improves risk profile of patients with acute coronary syndrome: A randomized crossover trial. Nutrients.

[28] Becatti M., Mannucci A., Barygina V., Mascherini G., Emmi G., Silvestri E., Wright D., Taddei N., Galanti G., Fiorillo C. (2017). Redox status alterations during the competitive season in élite soccer players: Focus on peripheral leukocyte-derived ROS. Intern. Emerg. Med..

[29] Fiorillo C., Becatti M., Attanasio M., Lucarini L., Nassi N., Evangelisti L., Porciani M., Nassi P., Gensini G., Abbate R. (2010). Evidence for oxidative stress in plasma of patients with Marfan syndrome. Int. J. Cardiol..

[30] Mehdizadehkashi A., Rokhgireh S., Tahermanesh K., Eslahi N., Minaeian S., Samimi M. (2021). The effect of vitamin D supplementation on clinical symptoms and metabolic profiles in patients with endometriosis. Gynecol. Endocrinol..

[31] Società Italiana Nutrizione Umana (2014). LARN-Livelli di Assunzione di Riferimento di Nutrienti ed Energia per la Popolazione Italiana.

[32] Becatti M., Fucci R., Mannucci A., Barygina V., Mugnaini M., Criscuoli L., Giachini C., Bertocci F., Picone R., Emmi G. (2018). A Biochemical Approach to Detect Oxidative Stress in Infertile Women Undergoing Assisted Reproductive Technology Procedures. Int. J. Mol. Sci..

[33] Laganà A.S., Salmeri F.M., Ban Frangež H., Ghezzi F., Vrtačnik-Bokal E., Granese R. (2019). Evaluation of M1 and M2 macrophages in ovarian endometriomas from women affected by endometriosis at different stages of the disease. Gynecol. Endocrinol..

[34] Shah D.K., Correia K.F., Vitonis A.F., Missmer S.A. (2013). Body size and endometriosis: Results from 20 years of follow-up within the Nurses’ Health Study II prospective cohort. Hum. Reprod..

[35] Goetz L.G., Mamillapalli R., Taylor H.S. (2016). Low body mass index in endometriosis is promoted by hepatic metabolic gene dysregulation in mice. Biol. Reprod..

[36] Ross R., Neeland I.J., Yamashita S., Shai I., Seidell J., Magni P., Santos R.D., Arsenault B., Cuevas A., Hu F.B. (2020). Waist circumference as a vital sign in clinical practice: A Consensus Statement from the IAS and ICCR Working Group on Visceral Obesity. Nat. Rev. Endocrinol..

[37] Schink M., Konturek P.C., Herbert S.L., Renner S.P., Burghaus S., Blum S., Fasching P.A., Neurath M.F., Zopf Y. (2019). Different nutrient intake and prevalence of gastrointestinal comorbidities in women with endometriosis. J. Physiol. Pharmacol..

[38] Missmer S.A., Chavarro J.E., Malspeis S., Bertone-Johnson E.R., Hornstein M.D., Spiegelman D., Barbieri R.L., Willett W.C., Hankinson S.E. (2010). A prospective study of dietary fat consumption and endometriosis risk. Hum. Reprod..

[39] Heard M.E., Melnyk S.B., Simmen F.A., Yang Y., Pabona J.M., Simmen R.C. (2016). High-Fat Diet Promotion of Endometriosis in an Immunocompetent Mouse Model is Associated with Altered Peripheral and Ectopic Lesion Redox and Inflammatory Status. Endocrinology.

[40] Hernáez Á., Castañer O., Goday A., Ros E., Pintó X., Estruch R., Salas-Salvadó J., Corella D., Arós F., Serra-Majem L. (2017). The Mediterranean Diet decreases LDL atherogenicity in high cardiovascular risk individuals: A randomized controlled trial. Mol. Nutr. Food Res..

[41] Polak G., Barczyński B., Kwaśniewski W., Bednarek W., Wertel I., Derewianka-Polak M., Kotarski J. (2013). Low-density lipoproteins oxidation and endometriosis. Mediat. Inflamm..

[42] Ross R. (1999). Atherosclerosis—An inflammatory disease. N. Engl. J. Med..

[43] Herrington W., Lacey B., Sherliker P., Armitage J., Lewington S. (2016). Epidemiology of atherosclerosis and the potential to reduce the global burden of atherothrombotic disease. Circ. Res..

[44] Burchardt P., Żurawski J., Żuchowski B., Kubacki T., Murawa D., Wiktorowicz K., Wysocki H. (2013). Low-density lipoprotein, its susceptibility to oxidation and the role of lipoprotein-associated phospholipase A2 and carboxyl ester lipase lipases in atherosclerotic plaque formation. Arch. Med. Sci..

[45] Tinelli C., Di Pino A., Ficulle E., Marcelli S., Feligioni M. (2019). Hyperhomocysteinemia as a Risk Factor and Potential Nutraceutical Target for Certain Pathologies. Front. Nutr..

[46] Proctor M.L., Murphy P.A. (2001). Herbal and dietary therapies for primary and secondary dysmenorrhea. Cochrane Database Syst. Rev..

[47] Laganà A.S., Sofo V., Salmeri F.M., Palmara V.I., Triolo O., Terzić M.M., Patrelli T.S., Lukanovic A., Bokal E.V., Santoro G. (2015). Oxidative Stress during Ovarian Torsion in Pediatric and Adolescent Patients: Changing The Perspective of The Disease. Int. J. Fertil. Steril..

[48] Amini L., Chekini R., Nateghi M.R., Haghani H., Jamialahmadi T., Sathyapalan T., Sahebkar A. (2021). The Effect of Combined Vitamin C and Vitamin E Supplementation on Oxidative Stress Markers in Women with Endometriosis: A Randomized, Triple-Blind Placebo-Controlled Clinical Trial. Pain Res. Manag..

[49] O’Connor J.M. (2001). Trace elements and DNA damage. Biochem. Soc. Trans..

[50] Messalli E.M., Schettino M.T., Mainini G., Ercolano S., Fuschillo G., Falcone F., Esposito E., Di Donna M.C., De Franciscis P., Torella M. (2014). The possible role of zinc in the etiopathogenesis of endometriosis. Clin. Exp. Obstet. Gynecol..

[51] Li Y., Zhong X., Cheng G., Zhao C., Zhang L., Hong Y., Wan Q., He R., Wang Z. (2017). Hs-CRP and all-cause, cardiovascular, and cancer mortality risk: A meta-analysis. Atherosclerosis.

[52] Ray K., Fahrmann J., Mitchell B., Paul D., King H., Crain C., Cook C., Golovko M., Brose S., Golovko S. (2015). Oxidation-sensitive nociception involved in endometriosis-associated pain. Pain.

[53] Maddern J., Grundy L., Castro J., Brierley S.M. (2020). Pain in Endometriosis. Front. Cell. Neurosci..

[54] Uno K., Nicholls S.J. (2010). Biomarkers of inflammation and oxidative stress in atherosclerosis. Biomark. Med..

[55] Forstermann U., Xia N., Li H. (2017). Roles of Vascular Oxidative Stress and Nitric Oxide in the Pathogenesis of Atherosclerosis. Circ. Res..

[56] Matsuoka H. (2001). Endothelial dysfunction associated with oxidative stress in human. Diabetes Res. Clin. Pract..

[57] Siekmeier R., Grammer T., Marz W. (2008). Roles of oxidants, nitric oxide, and asymmetric dimethylarginine in endothelial function. J. Cardiovasc. Pharmacol. Ther..

[58] Dai J., Jones D.P., Goldberg J., Ziegler T.R., Bostick R.M., Wilson P.W., Manatunga A.K., Shallenberger L., Jones L., Vaccarino V. (2008). Association between adherence to the Mediterranean diet and oxidative stress. Am. J. Clin. Nutr..

[59] Nirgianakis K., Egger K., Kalaitzopoulos D.R., Lanz S., Bally L., Mueller M.D. (2021). Effectiveness of Dietary Interventions in the Treatment of Endometriosis: A Systematic Review. Reprod. Sci..

[60] Ott J., Nouri K., Hrebacka D., Gutschelhofer S., Huber J., Wenzl R. (2012). Endometriosis and nutrition-recommending a Mediterranean diet decreases endometriosis-associated pain: An experimental observational study. J. Aging Res. Clin. Pract..

[61] Santoro L., D’Onofrio F., Campo S., Ferraro P.M., Tondi P., Campo V., Flex A., Gasbarrini A., Santoliquido A. (2012). Endothelial dysfunction but not increased carotid intima-media thickness in young European women with endometriosis. Hum. Reprod..

